# Performance measures of 8,169,869 examinations in the National Breast Cancer Screening Program in Taiwan, 2004–2020

**DOI:** 10.1186/s12916-023-03217-7

**Published:** 2023-12-15

**Authors:** Melissa Min-Szu Yao, Vu Pham Thao Vy, Tony Hsiu-Hsi Chen, Hsian-He Hsu, Giu-Cheng Hsu, Cindy S. Lee, Li-Ju Lin, Shu-Li Chia, Chao-Chun Wu, Wing P. Chan, Amy Ming-Fang Yen

**Affiliations:** 1 Department of Radiology, Koo Foundation Sun Yat-Sen Cancer Center, Taipei, 112 Taiwan; 2 International PhD Program of Medicine, Taipei Medical University, Taipei, 110 Taiwan; 3 Department of Radiology, Thai Nguyen National Hospital, Thai Nguyen, 24000 Vietnam; 4 YRDx-AI Lab, Ho Chi Minh City, 70000 Vietnam; 5 Institute of Epidemiology and Preventive Medicine, College of Public Health, National Taiwan University, Taipei, 100 Taiwan; 6 Department of Radiology, Tri-Service General Hospital and National Defense Medical Center, Taipei, 11490 Taiwan; 7 Department of Radiology, Kang-Ning General Hospital, Taipei, 114 Taiwan; 8 Department of Radiology, State University of New York, Stony Brook, NY 11794 USA; 9 Health Promotion Administration, Ministry of Health and Welfare, Taipei, 103 Taiwan; 10 Department of Radiology, Wan Fang Hospital, Taipei Medical University, 111 Xinglong Road, Section 3, Taipei, 116 Taiwan; 11 Department of Radiology, School of Medicine, College of Medicine, Taipei Medical University, Taipei, 110 Taiwan; 12 School of Oral Hygiene, College of Medicine, Taipei Medical University, Taipei, 110 Taiwan

**Keywords:** Asia, Breast cancer, Mammography, Screening, Survival rate

## Abstract

**Background:**

The benefits of mammographic screening have been shown to include a decrease in mortality due to breast cancer. Taiwan’s Breast Cancer Screening Program is a national screening program that has offered biennial mammographic breast cancer screening for women aged 50–69 years since 2004 and for those aged 45–69 years since 2009, with the implementation of mobile units in 2010. The purpose of this study was to compare the performance results of the program with changes in the previous (2004–2009) and latter (2010–2020) periods.

**Methods:**

A cohort of 3,665,078 women who underwent biennial breast cancer mammography screenings from 2004 to 2020 was conducted, and data were obtained from the Health Promotion Administration, Ministry of Health and Welfare of Taiwan. We compared the participation of screened women and survival rates from breast cancer in the earlier and latter periods across national breast cancer screening programs.

**Results:**

Among 3,665,078 women who underwent 8,169,869 examinations in the study population, the screened population increased from 3.9% in 2004 to 40% in 2019. The mean cancer detection rate was 4.76 and 4.08 cancers per 1000 screening mammograms in the earlier (2004–2009) and latter (2010–2020) periods, respectively. The 10-year survival rate increased from 89.68% in the early period to 97.33% in the latter period. The mean recall rate was 9.90% (95% CI: 9.83–9.97%) in the early period and decreased to 8.15% (95%CI, 8.13–8.17%) in the latter period.

**Conclusions:**

The evolution of breast cancer screening in Taiwan has yielded favorable outcomes by increasing the screening population, increasing the 10-year survival rate, and reducing the recall rate through the participation of young women, the implementation of a mobile unit service and quality assurance program, thereby providing historical evidence to policy makers to plan future needs.

**Supplementary Information:**

The online version contains supplementary material available at 10.1186/s12916-023-03217-7.

## Background

Breast cancer is the most common cancer among women and the fifth leading cause of mortality worldwide, accounting for 2.3 million new cases, 11.7% of all cancers in women, and 685,000 deaths (6.9%) in 2020 [1]. The incidence of breast cancer is rising rapidly in transitioning countries in South America, Africa, and Asia. In Asia, breast cancer incidence has doubled or tripled in Japan, Korea, Hong Kong, Singapore, and Taiwan over the past 40 years [2–4].

Across various study designs, the benefits of mammographic screening have been shown to include a decrease in mortality due to breast cancer, lower treatment morbidity, and an increase in life expectancy. In a randomized controlled trial of 40- to 64-year-old women enrolled in the Health Insurance Plan of Greater New York, Shapiro et al. [5] reported that compared to controls, screening resulted in a 25% lower breast cancer mortality rate among women aged 40–49 years and those aged 50–59 years. Lee et al. [6] reported that, compared to other screening strategies used in the USA, annual mammography starting at 40 years of age maximizes life-extending benefits for women.

In Taiwan in 2018, the prevalence of breast cancer was 188–194 per 100,000 women aged 45–69 years. That year, new cases totaled 16,988 (including invasive cancer and ductal carcinoma in situ), and the number of deaths reached 2418. In 2020, the age-standardized incidence rate was 47.8 per 100,000 and the death rate was 13.6 per 100,000 [7]. Starting in 2004, Taiwan’s government implemented the population-based National Breast Cancer Screening Program through the Health Promotion Administration (HPA), providing free screening for breast cancer by performing mammograms biennially for women aged 50–69 years [8]. Beginning in December 2009, the lower age limit was modified to 45 years. Beginning in 2010, women aged 40–44 years with second-degree relatives diagnosed with breast cancer were included. Initially, screening mammograms were provided using a unit fixed in a hospital. However, in 2010, a mobile unit was added. The purpose of this study was to assess the outcome measures, the incidence of breast cancer detection, and survival rates, due to the evolution of policymaking for breast cancer screening programs in Taiwan between 2004 and 2020.

## Methods

This study was approved by the Taipei Medical University Joint Institutional Review Board (N202112050), and written informed consent was waived due to the retrospective nature of this study.

### Data source and study population

**Fig. 1:**
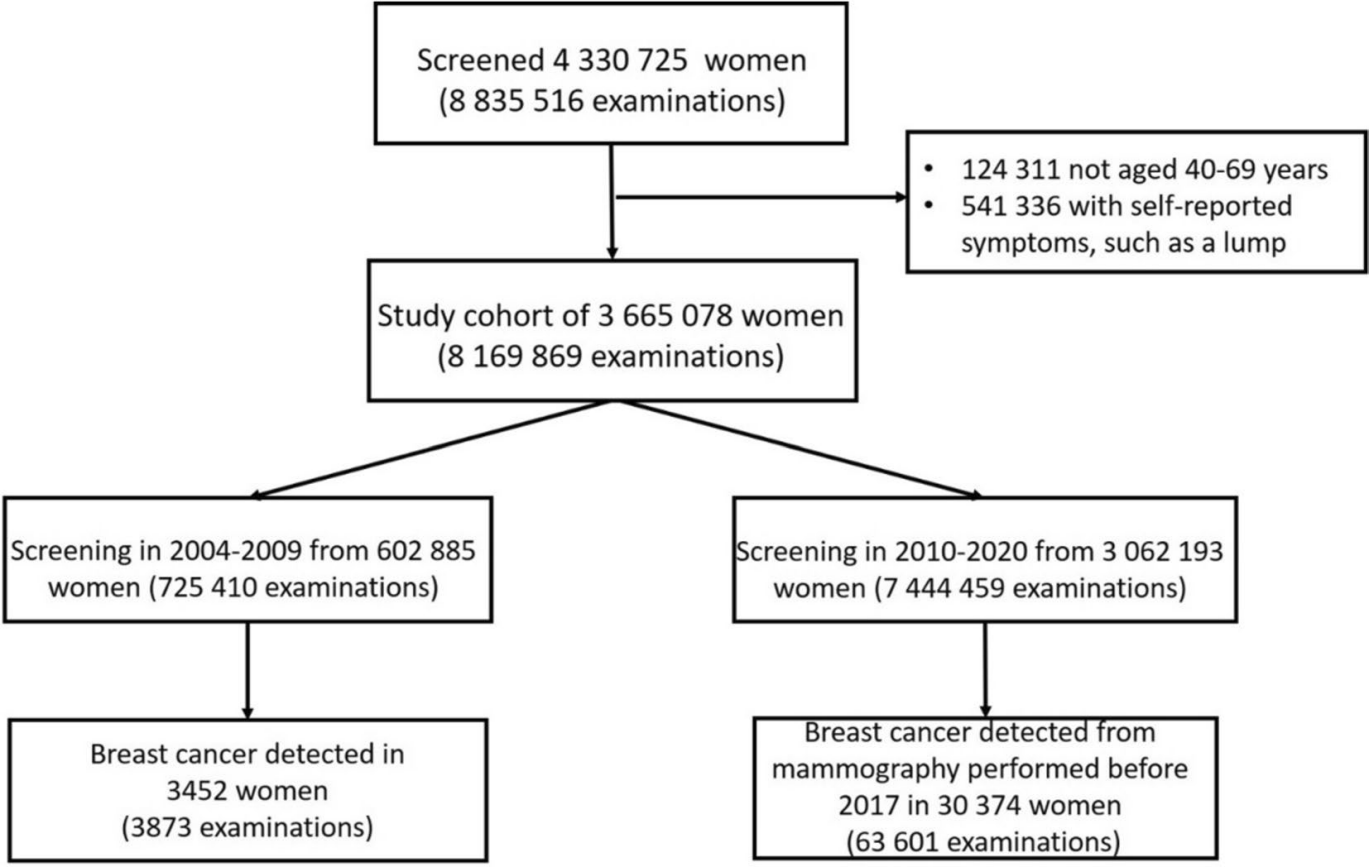
Study flowchart. Screening mammograms performed between 2004 and 2020 were selected based on the given exclusion criteria. Starting in 2010, a mobile unit was deployed, and the age range was widened to include younger women

Data for this cohort study were sourced from biennial breast cancer mammography screenings performed as part of the Taiwan government-run HPA of the Health Promotion Administration, Ministry of Health and Welfare, Taiwan. Data were prospectively collected according to government policies. Records were linked to the Taiwan Cancer Registry to validate breast cancer diagnoses and to identify breast cancers detected outside the screening program. Examinations for this study were drawn from 8,835,516 examinations of women who underwent at least one mammographic screening examination between 2004.7.1 and 2020.12.31 (Fig. 1). Included in the database but excluded from our study were examinations of women aged less than 40 years or more than 69 years (*n* = 124,311) and those with self-reported symptoms (*n* = 541,336) such as a lump. Therefore, 8,169,869 examinations in 3,665,078 women were included in this study.

### Mammographic data collection

All participants were requested to complete a questionnaire at each visit reporting menopausal status, reproductive history, personal history of breast diseases, family history of breast cancer, self-reported symptoms, and history of breast biopsies. All mammograms were interpreted by qualified radiologists using the BI-RADS assessment and recommendation categories as follows: 0, need additional imaging evaluation; 1, negative; 2, benign; 3, probably benign; 4, various degrees of suspicion; and 5, highly suggestive of malignancy. The definitions of all metrics were taken from the Breast Imaging Reporting & Data System Breast Imaging Reporting & Data System(ACR BI-RADS) 4th edition during the years 2004 through 2012 and the 5th edition in all subsequent years [9]. To abide with the requirements of the Taiwan Radiological Society, only qualified radiologists were used to make these interpretations. Details regarding data collection and training of radiologists and radiographers are provided in Additional file 1: Text S1.

### Performance metrics

In performing the double-check, our experts used the ACR BI-RADS 5th edition to complete a series of performance measures [10]: recall rate, cancer detection rate (CDR), rates for three positive predictive values (PPVs), sensitivity, and specificity. The PPVs were PPV1, indicating that, at the first assessment, the mammogram was categorized as 0, 3, 4, or 5; PPV2, indicating that a biopsy was recommended; and PP3, indicating that a biopsy was performed. If breast cancer was diagnosed as positive (final assessment of 4 or 5) on mammography within 12 months, screen-detected breast cancer was determined (a true positive finding). The accuracy of mammography screening is usually assessed during a one-year follow-up period [11]. Sensitivity was determined by dividing the number of true positive examinations by the total number of examinations associated with cancer, and specificity was determined by dividing the number of true negative examinations by the total number of examinations without cancer [12]. These true positives contributed to the numerator of CDR and PPV rates. In contrast, false negatives occurred when a mammogram was initially assessed as negative (categories 1 and 2), but within 12 months, the patient was diagnosed with cancer. Data were divided into two periods based on the protocol for screening eligibility (2004–2009 and 2010–2017), and these two periods were independently analyzed. Survival times were calculated using the date of breast cancer diagnosis to the date of death or the end of 2019, whichever occurred first.

### Statistical analysis

The rates of screen-detected and histology-proven breast cancers were compared according to age, family history, menopausal status, and breast density. Chi-square tests were performed to compare categorical variables between the groups. Performance measures were determined as percentages and 95% confidence intervals (CIs), using the Wald asymptotic method.

Benchmarks were derived across the two periods using the distributions of mammographic performance metrics achieved by the interpreting radiologists. The medians for the interpreting radiologists were found, and quartiles were used as measures of variability. Interquartile ranges represent the middle half of the radiologists.

Survival curves were generated using the Kaplan–Meier method, and the log-rank test was used to determine survival differences across a given period. All statistical analyses were performed using SAS version 9.4 (SAS Institute, Inc., Cary, NC, USA).

## Results

### Trends in mammographic screening and survival rates

**Fig. 2:**
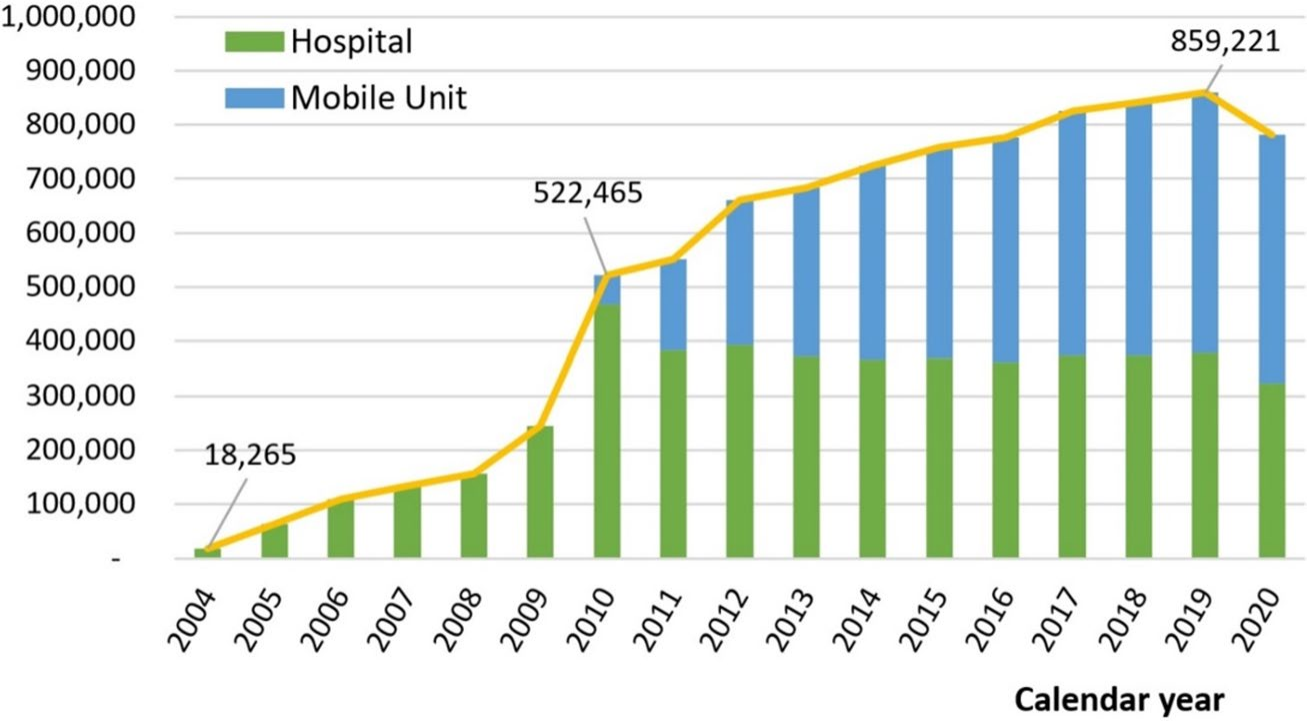
Volume of mammograms performed as part of the population-based mammographic screening program in Taiwan 2004–2020

Between 2004 and 2020, 8,169,869 screening mammograms qualified for our study were performed across 3,665,078 women. The screened population increased from 18,265 (3.9% of all women) in 2004 to 859,221 (approximately 40% of all women) in 2019. The number of qualified screenings grew by an average of 28.75% annually over the 16 years in the study period. Actual annual growth was gradual near the inception of the program (2004); however, once the mobile unit was implemented and the age requirement was lowered (both in 2010), growth was much greater (Fig. 2). Since 2015, screenings performed in the mobile unit have outnumbered those performed at brick-and-mortar hospitals; they comprised 480,456 of the 859,221 examinations (55.9%) performed in 2019. In 2020, the total number of mammograms dropped owing to the COVID-19 pandemic.

The proportion of screening mammograms performed in women aged 50–69 years was 98.7% in the earlier period and 63.3% in the latter period (Table 1). Narrowing the age group to 50–65 years, the proportions were 84.9% and 54.2%, respectively. Conversely, the mammograms performed on young women (aged 40–49 years) comprised 1.3% and 36.7% of the total, respectively. During the latter period, the mobile unit served more senior women than the hospitals (Table 1 and Additional file 2: Table S1). Among young women, breast cancer was detected at a rate of 1.8% of the total group in the earlier period compared to 21.4% in the latter period. In contrast, these figures were 83.3% and 64.3%, respectively, among the group aged 50 to 65 years. In the latter period, approximately 10.1% of women with breast cancer had a family history of breast cancer. Comparatively, 7.1% of cancer-free women had a family history of breast cancer (Table 1). The breasts in which cancer was detected were denser than those in which cancer was not detected (*P* < 0.001).

**Table 1:** Clinical demographics for mammographic screening examinations

Characteristic	2004–2009				2010–2020			
	**No**	**%**	**No. with SDC**	**%**	**No**	**%**	**No. with SDC**	**%**
**No. of women screened**	**602,885**		**3452**		**3,062,193**		**30,374**	
**Age at screening**^**a**^**/diagnosis, y**								
40–44	1100	**0.2**	9	**0.3**	28,284	**0.9**	138	**0.5**
45–49	6815	**1.1**	53	**1.5**	1,096,190	**35.8**	6054	**19.9**
50–54	237,249	**39.4**	1165	**33.7**	659,752	**21.5**	6502	**21.4**
55–59	171,544	**28.4**	1032	**29.9**	566,360	**18.5**	6615	**21.8**
60–64	102,779	**17.1**	681	**19.7**	433,861	**14.2**	6392	**21.0**
65–69	83,398	**13.8**	512	**14.8**	277,746	**9.1**	4673	**15.4**
**Family history of breast cancer**								
No	570,496	**94.6**	3150	**91.3**	2,844,288	**92.9**	27,299	**89.9**
Yes	32,389	**5.4**	302	**8.7**	217,905	**7.1**	3075	**10.1**
**No. of screenings**	**725,410**		**3452**		**7,444,459**		**30,374**	
**Screening service**								
Prevalence screenings	602,885	**83.1**	3051	**88.4**	2,477,060	**33.3**	13,184	**43.4**
Subsequent screenings	122,525	**16.9**	401	**11.6**	4,967,399	**66.7**	17,190	**56.6**
**Menopausal status**								
Premenopausal	99,316	**13.7**	554	**16.0**	2,164,086	**29.1**	9216	**30.3**
Postmenopausal	626,094	**86.3**	2898	**84.0**	5,280,373	**70.9**	21,158	**69.7**
**Breast density**								
Fatty breast	83,970	**11.6**	253	**7.3**	310,759	**4.2**	749	**2.5**
Scattered fibroglandular density	254,468	**35.1**	1098	**31.8**	1,655,441	**22.2**	6019	**19.8**
Heterogeneously dense	335,543	**46.3**	1888	**54.7**	4,169,452	**56.0**	19,118	**62.9**
Extremely dense	51,415	**7.1**	213	**6.2**	1,308,619	**17.6**	4486	**14.8**

*SDC* screen-detected cancer

^‡^*P* < 0.001 (chi-square test) for all comparisons between periods

^§^*P* < 0.001 (chi-square tests) for all comparisons between women with SDCs and cancer-free women

^a^Age at first screening

**Fig. 3:**
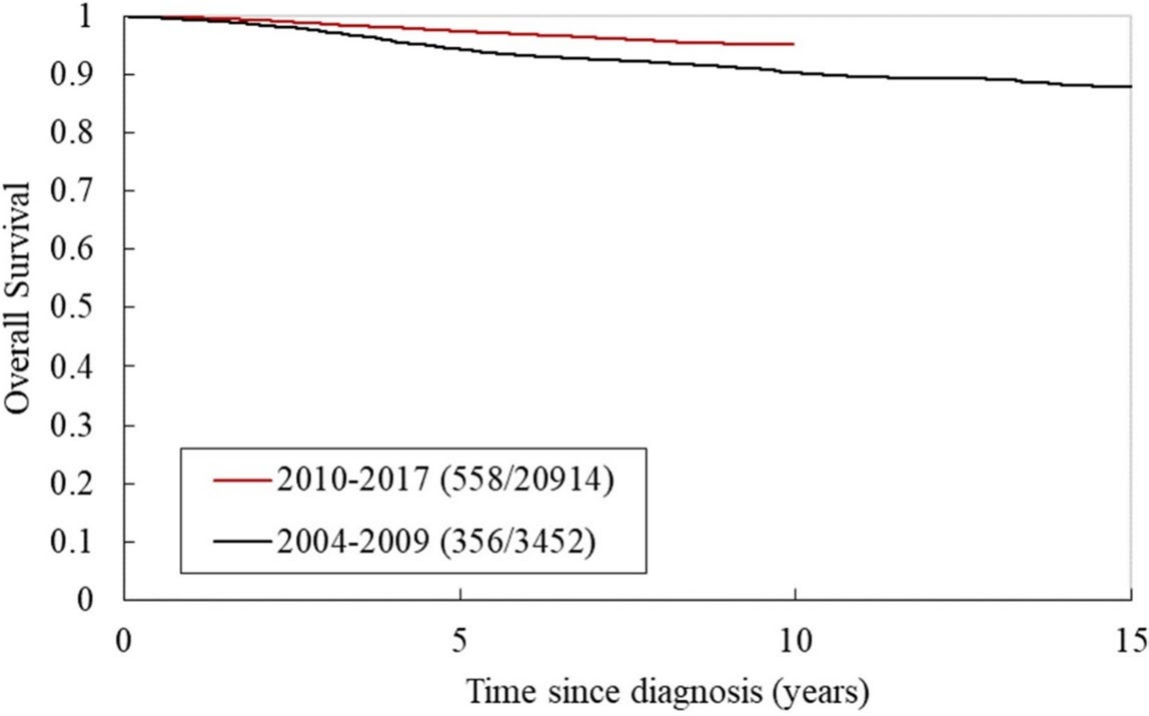
Overall survival rates among women with breast cancer across two time periods

Figure 3 shows the Kaplan–Meier curve for 10-year survival across 3,452 women from 2004 to 2009 and 20,914 women from 2010 to 2017 (log-rank test, *P* < 0.001). During follow-up, the numbers of deaths were 356 and 558, respectively*.* Between the two periods, the survival rate was significantly different for several age groups: *P* = 0.00316 for those aged 45–49 years and *P* ≤ 0.0001 for all other groups (50–54, 55–59, 60–64, and 65–69 years; Additional file 3: Fig. S1).

From 2004 through 2009, the mean recall rate was 9.90% (95% CI, 9.83–9.97%), but from 2010 to 2020, it was 8.15% (95% CI, 8.13–8.17%) (Table 2). During the latter period, the rate was higher in hospitals (8.99%; 95% CI, 8.96–9.01%) than in the mobile unit (7.30%; 95% CI, 7.27–7.72%). Of 7,444,459 screenings performed in the latter period, 30,374 resulted in positive breast cancer diagnoses (final assessment with BI-RADS 4 or 5); therefore, the CDR was 4.08 (95% CI, 4.03, 4.13) per 1000 screenings. The portion representing the greatest CDR (4.88; 95% CI, 4.81–4.95) was collected in hospitals in the latter period. This was followed by the portion collected in the earlier period (4.76; 95% CI, 4.60–4.92) and the portion collected in the mobile unit (3.26; 95% CI, 3.20–3.32). The same pattern was observed for PPV1:5.43% (95% CI, 5.36–5.51%) among those collected at hospitals in the latter period, 4.81% (95% CI, 4.65–4.96%) among those collected in the earlier period, and 4.47% (95% CI, 4.39–4.54%) among those collected in the mobile unit. Among those mammograms categorized as 4 or 5, the PPV2 rate was 34.46% (95% CI, 33.53–35.39%) in the earlier period and 27.19% (95% CI, 26.93–27.45%) in the latter period, broken down into hospital-collected mammograms 26.18% (95% CI, 25.85–26.91%) and mobile-unit-collected mammograms 28.89% (95% CI, 28.45–29.32%) (Table 2).

**Table 2:** Performance measures of digital mammographic screenings for breast cancer^a^

Measure	2004–2009	2010–2020		
	**Fixed-unit**	**Hospital**	**Mobile**	**Subtotal**
**Recall rate, %**	**9.90 (9.83, 9.97)**	**8.99 (8.96, 9.01)**	**7.30 (7.27, 7.32)**	**8.15 (8.13, 8.17)**
Total no. of examinations	725,410	3,762,883	3,681,576	7,444,459
No. of abnormal interpretations	71,819	338,101	268,693	606,794
**CDR per 1000 examinations, No**	**4.76 (4.6, 4.92)**	**4.88 (4.81, 4.95)**	**3.26 (3.20, 3.32)**	**4.08 (4.03, 4.13)**
No. detecting cancer	3452	18,372	12,002	30,374
Total no. of examinations	725,410	3,762,883	3,681,576	7,444,459
**PPV1, abnormal interpretations, %**	**4.81 (4.65, 4.96)**	**5.43 (5.36, 5.51)**	**4.47 (4.39, 4.54)**	**5.01 (4.95, 5.06)**
No. of mammograms detecting cancer	3452	18,372	12,002	30,374
Initial BI-RADS category of 0, 3, 4, or 5	71,819	338,101	268,693	606,794
**PPV2, biopsy recommended, %**	**34.46 (33.53, 35.39)**	**26.18 (25.85, 26.51)**	**28.89 (28.45, 29.32)**	**27.19 (26.93, 27.45)**
No. of mammograms detecting cancer	3452	18,372	12,002	30,374
Final BI-RADS category of 4 or 5	10,017	70,176	41,546	111,722
**PPV3, biopsy performed, %**	**55.08 (53.85, 56.31)**	**34.70 (34.3, 35.11)**	**37.20 (36.67, 37.73)**	**35.65 (35.33, 35.97)**
No. of mammograms detecting cancer	3452	18,372	12,002	30,374
Final BI-RADS category of 4 or 5 with biopsy	6267	52,939	32,262	85,201
**Sensitivity, %**^**b**^				
**1-year follow-up**	83.28 (82.18,84.38)	84.02 (83.46,84.57)	84.40 (83.68,85.12)	84.16 (83.72,84.59)
True positive	3685	14,177	8179	22,356
False negative	740	2697	1512	4209
**2-year follow-up**	73.28 (72.1,74.47)	73.61 (73.01,74.22)	72.96 (72.17,73.75)	73.37 (72.89,73.85)
True positive	3928	15,082	8801	23,883
False negative	1432	5406	3262	8668
**Specificity, %**^**b**^				
**1-year follow-up**	90.55 (90.48,90.62)	91.35 (91.31,91.38)	92.90 (92.87,92.93)	92.05 (92.03,92.08)
True negative	652,851	2,532,504	2,146,716	4,679,220
False positive	68,134	239,937	164,063	404,000
**2-year follow-up**	90.57 (90.5,90.64)	91.37 (91.33,91.4)	92.92 (92.89,92.95)	92.07 (92.05,92.1)
True negative	652,159	2,529,795	2,144,966	4,674,761
False positive	67,890	239,032	163,441	402,473

*CDR* cancer detection rate, *PPV* positive predictive value

^†^*P* < 0.001 (chi-square tests) for all comparisons between time periods and collection locations (hospital vs. mobile unit)

^a^Numbers in parentheses are 95% CIs

^b^Sensitivity and specificity only counts those screened before 2017

### Cancers detected in screening mammograms

In the mammograms collected from 2010 to 2017, 20,914 indicated cancer, 2150 (10.28%) were histologically shown to be ductal carcinoma in situ (DCIS), and 18,764 (89.72%) were shown to be invasive. Of the invasive cancers, 20.02% were 10 mm or smaller, 29.58% were between 11 and 20 mm, and approximately 20.98% were > 20 mm at the time of diagnosis (details are provided in Additional file 4: Table S2 and Additional file 5: Table S3).

### Mobile unit performance

The volume of screening mammograms performed in the mobile unit appeared to have increased the number of people reached by this program (Fig. 1). Since its deployment, the mobile unit has performed 49.45% of all screening mammograms (3,681,576 of 7,444,459; Table 2). From 2010 to 2020, 12,002 cancers were detected by the mobile unit, comprising 39.51% of all cancers detected in that period. Comparatively, 3452 cancers were detected before the mobile unit was deployed (Table 2).

### Performance between radiologists

In total, 739 radiologists interpreted breast cancer screenings from 2004 through 2020. The demand for radiologists for this task increased by 3.28 times, from 134 in 2004 to 439 in 2020. The details are provided in Additional file 6: Fig. S2, Additional file 7: Tables S4, and Additional file 8: Table S5.

## Discussion

Taiwan’s Breast Cancer Screening Program is a population-based mass screening program that has offered biennial mammographic breast cancer screening for women aged 50–69 years since 2004 and for those aged 45–69 years since 2009. In addition, beginning in 2010, women aged 40–44 years with second-degree relatives diagnosed with breast cancer were included. After the implementation of a mobile unit and including younger eligible women, the screened population increased from 3.9 to 40% of all women in the population from 2004 to 2019. The mammographic screening rate in Taiwan is increasing, which is consistent with those reported elsewhere [4, 13]. We also found that mammography screening was associated with an increase in survival rates. Breast cancer was detected in 4.76 per 1000 examinations in the earlier period (2004–2009), whereas it was 4.08 per 1000 examinations in the latter period (2010–2020). The survival rate increased from 89.68% in the early period to 97.33% in the latter period. This increase is due to improved survival rates for patients with aggressive breast cancer, especially those with moderate or poor prognosis. Recent advances in targeted therapy have provided more specific and effective therapeutic options for breast cancer treatment [14]. Target therapy aims to inhibit specific molecules that promote tumor growth and survival [15–17]. In contrast, immune therapies have emerged as promising targeted therapies for patients with triple-negative breast cancer [18, 19].

According to the American Cancer Society [20], early detection of breast cancer (in the localized stage) is associated with a 5-year relative survival rate of 99%. In Europe, every woman aged 50 to 69 years joins a mammographic screening program; the actual coverage is 49% in the east, 62% in the west, 64% in the north, and 69% in the south [21]. Zielonke et al. [21] estimated that this program prevents 34% of potential breast-cancer-specific deaths across Europe. In 2019, 76.4% of women aged 50–74 years had mammograms within the past two years in the US [22]. The US Preventive Services Task Force recommends that women start annual screening at the age of 40 [23], but the American Cancer Society recommends that women at average risk begin annual screening at 45 years of age [6]. The US Preventive Services Task Force reported that in a sample of 10,000 women, screening reduced mortality over a 10-year period by 8% and 21% for those aged 50–59 and 60–69 years, respectively [24]. If breast cancer is detected early, there are more treatment options with less invasive therapy and a better chance of survival. Our study suggests that implementing a larger breast cancer screening program with a lower screening age has improved the detection of breast cancer and increased the survival time of patients. It should be noted that the reduction in mortality associated with breast cancer depends on several factors, including the stage of diagnosis, breast cancer subtypes, tumor aggressiveness and treatment strategies that will be investigated in future studies.

Comparing the hospital-collected mammograms with those collected in the mobile unit, the mean recall rate was greater in the former (8.99% vs. 7.3%). While both of these are within the normal range recommended by the ACR (5–12%) [12, 25], the combined rate for this period was lower than that in the earlier period before the mobile unit was deployed. The CDR was not significantly different between the mammograms collected at the hospital and those collected in the mobile unit (4.88 vs. 3.26 per 1000). In addition, all the 1-year interval cancers were calculated as false negatives, with 740 and 4209 cases, resulting in 83.28% (95%CI, 82.18–84.38%) and 84.16% (95%CI, 83.72–84.59%) in the early and latter periods, respectively. This means that the sensitivity estimates are likely to be higher, with significantly more interval cancers in the 1–2 years than in the 0–1 years. In the latter period, the sensitivity and specificity of the 1- and 2-year intervals were 84.16% and 92.05% for 1-year, and 73.37% and 92.07% for 2-year intervals, respectively (Table 2).

Implementation of the mobile unit increased the coverage of the screening program across the population. According to our data, the PPV2 and PPV3 rates grew closer once the mobile unit was deployed. As a result, a larger proportion of women were recommended for biopsy in the more recent period. Because biopsy procedures have improved, women now have more options, such as vacuum-assisted biopsy, incisional biopsy through a small wound that does not leave a scar, and stereotactic needle localization excisional biopsy. Screening mammography is the primary imaging modality for the early detection of breast cancer. During our study period, the rate of DCIS detection increased, and the proportion of invasive cancers decreased over time. Furthermore, minimal cancers were detected more frequently over time, whereas the proportion of metastatic cancers decreased from 1.47 to 0.78%. These data show that consecutive breast cancer screenings can aid in the early detection of breast cancer.

The mean age at which cancer was first detected decreased from 57.3 years to 55.5 years among women utilizing hospitals for their mammograms. For those using the mobile unit, the mean age at first detection was 56.6 years. Premenopausal women accounted for 13.7% of those screened in the earlier period but 30.3% of those screened in the latter period. Women aged 40–49 years accounted for 1.3% of those screened in the earlier period but 21.6% of those screened in the latter period. The details are provided in Additional file 9: Table S6 and Additional file 10: Fig. S3. Young women preferred screening in hospitals, resulting in a greater proportion of screen-detected cancers (SDCs) among those attending hospitals (49.4%) than among those attending mobile units (34.3%). Duffy et al. [26] reported a statistically significant 25% decrease in mortality during a 10-year follow-up of those aged 40–49 years when annual mammographic screening was performed. Some studies have suggested that massive screening with mammography could reduce mortality [26–28], especially when starting at 40 years of age [26, 28–30]. A significant reduction in mortality (RR, 0.75; 95% CI, 0.58–0.97) over the first 10 years of screening was reported when screening began at 40 years of age [30]. Taken together, these studies and our data support the policy of beginning mammographic screening at 40 years of age and the continuation of this policy.

The mobile unit provides a convenient option for women participating in early cancer detection programs. This helped lower the barriers to attaining breast cancer screenings, thus increasing the number of mammographic screenings. In the mobile unit, 26.2% of the mammograms performed from 2010 to 2020 were prevalence screening. As a result, the mobile unit was responsible for rapidly increasing the number of SDCs from 3452 before their deployment to 30,374 after their deployment (12,002 were from mammograms collected in the mobile unit). Furthermore, 34.3% of the prevalence screenings collected in the mobile unit yielded SDCs. The mobile unit not only helped resolve problems with crowds waiting for mammograms, but it was also more convenient for women [30–32]. Across the entire study period, 5,860,437 mammograms were performed on women aged 40–59 years. In Brazil, a program utilizing mobile screening units screened 122,634 women with a cumulative coverage rate of 54.8% in the target population [33]. Furthermore, a retrospective study conducted in France showed that mobile mammography units can reduce social and territorial inequalities [34].

This study has several limitations. First, mammographic devices such as film screens, computer radiography (CR), and digital radiography (DR) have not yet been investigated. From 2004 to 2009, film screens were the most popular device; however, DR became the most popular device after 2010. As a result, breast cancer may have been overlooked before 2010. Some CR devices could have remained in use since 2010, particularly in mobile units. Second, before 2010, women were not asked to self-report their symptoms; therefore, the percentage of women with self-reported symptoms was very small. Third, the lack of data makes it impossible to interpret the full benefits of the mobile mammography unit. For example, it can reduce social and territorial inequality. Fourth, our results only included cases detected within 1 year after screening; women who developed breast cancer between 12–24 months after screening mammography with positive results will not be included. The Health Promotion Bureau has set a performance indicator for referring patients within three months. The majority of true positives should have been detected within one year. In addition, this one-year follow-up period is usually used to calculate the accuracy of mammography screening and to monitor additional imaging over the 12 months of screening examination [11, 12].

## Conclusions

The evolution of breast cancer screening has achieved favorable outcomes in Taiwan, particularly the right strategy for the implementation of a mobile unit service to elevate screened women and include a younger population to improve the early detection and survival rates of breast cancer. This result may provide a reference for other countries.

### Supplementary Information

**Additional file 1: Text S1.** Methods.

**Additional file 2: Table S1.** Clinical Demographics for Mammographic Screenings for Breast Cancer*.

**Additional file 3: Figure S1.** Overall Survival Rates in Women with Breast Cancer Across the Two Time Periods by Age Group. (a) 40-49, (b) 50-54, (c) 55-59, (d) 60-64, (e) 65-69.

**Additional file 4: Table S2.** Performance Measure for Mammographic Screenings Including Self-Reported Symptoms, 2010-2017.

**Additional file 5: Table S3.** Characteristics of Breast Cancers Detected using Digital Mammographic Screening.

**Additional file 6: Figure S2.** The number of qualified radiologists and radiographers involved in the nationwide mammography screening program from 2004-2020.

**Additional file 7: Tables S4.** Radiologists’ Performance Measures by Time Period.

**Additional file 8: Table S5.** Radiologists’ Performance Measures by Screening Year.

**Additional file 9: Table S6.** Performance Measures of Digital Mammographic Screenings for Breast Cancer in 2010-2020 by age group.

**Additional file 10: Figure S3.** Distribution of screening population followed by age in 2 periods.

## Data Availability

The datasets generated and/or analyzed during the current study are not publicly available due to restrictions based on regulations, but are available from the corresponding author on reasonable request.

## Abbreviations

95% CI: 95% Confidence intervals
BI-RADS: Breast Imaging Reporting & Data System
DCIS: Ductal carcinoma in situ
HPA: Health Promotion Administration
SDCs: Screen-detected cancers
