# Supplementary material for: Performance measures of 8,169,869 examinations in the National Breast Cancer Screening Program in Taiwan, 2004–2020
Source: BMC Med. 2023 Dec 15;21:497. doi: 10.1186/s12916-023-03217-7 (PMC10724902; doi:10.1186/s12916-023-03217-7)
Supplement: Supplementary file 1 — Additional file 1: Text S1. Methods. [file 12916_2023_3217_MOESM1_ESM.docx]

## Text S1. Methods

## Data Source

Data for this cohort study was sourced from the biennial mammographic screenings for breast cancer performed as part of Taiwan’s government-run HPA from the Health Promotion Administration, Ministry of Health and Welfare, Taiwan, between July 1, 2004 and December 31, 2020. It was prospectively collected following government policies. The costs for the screenings were fully reimbursed by the government, and the records, referrals, and diagnoses were electronically stored at the HPA-run Surveillance and Data Monitor Center. Records were linked to the Taiwan Cancer Registry to validate breast cancer diagnoses and identify breast cancers detected outside of the screening program. At the time of analysis, the Cancer Registry was complete only up to the end of 2018; screen-detected breast cancers for the years 2019 and 2020 were included by accessing the Surveillance and Data Monitor Center. Starting in 2004, all women aged 50 to 69 years were eligible for government-paid mammographic screening every two years. Women with family histories of breast cancer in first- or second-degree relatives became eligible at 40 years of age. In November 2009, the family history requirement was dropped for those aged at least 45 years.

## Mammographic Data Collection

Mammograms initially categorized as 0, 3, 4, or 5 were referred to HPA certified hospitals for further assessment, diagnosis, or treatment. In cases of category 0, the final assessment was determined from the additional imaging or testing. If the diagnosis was breast cancer, several descriptive characteristics were recorded: Ductal carcinoma in situ (DCIS) vs. invasive cancer, cancer size, whether the cancer was minimal, node status, and cancer stage. Based on the initial determinations of each radiologist, we chose images and reports from each year for our experts to double-check. This helped to assure that incidences of cancer were not missed and allowed us to obtain robust estimates. To abide by the requirements of the Taiwan Radiological Society, only qualified radiologists were used to make these interpretations. Qualified radiologists were those who interpreted at least 240 images per half year or 1000 over the prior two years. Board-certified radiologists were allowed to interpret mammograms if they completed both a mammogram interpretation or quality control education program and 10 educational credits each year. We also included well-trained mammography radiographers who interpreted at least 200 cases in a two-year period and earned a certificate of mammography training (40 hours) from the Taiwan Radiological Society. Finally, radiographers needed to maintain their qualifications by participating in mammography training courses organized by the agency commissioned by our Department of Radiology within the prior two years and passing the annual Health Department mammography qualification with a grade B or better. In addition, medical institutions were required to be certified in mammography, and any change in staff or mammographic instruments was expected to be reported to the department within two weeks.

## Performance metrics

We use some metrics for performance measures: recall rate, CDR, rates for three positive predictive values (PPVs), sensitivity, and specificity. The PPVs were PPV1, indicating that at first assessment, the mammogram was categorized as 0, 3, 4, or 5; PPV2, indicating that a biopsy was recommended; and PP3, indicating that a biopsy was performed. If breast cancer was diagnosed within 12 months of a positive (final assessment of 4 or 5) mammogram, screen-detected breast cancer was determined (a true positive finding). These true positives contributed to the numerator of the CDR and the PPV rates. On the other hand, false negatives (FNs) occurred when a mammogram was initially assessed as negative (category 1), but within 12 months, the patient was diagnosed with cancer. Data were divided into two periods based on the protocol for screening eligibility (2004-2009 and 2010-2017), and these two time periods were independently analyzed. Survival times were calculated using the date of diagnosis and the date of death or the end of 2019, whichever occurred first.
