# Supplementary material for: Performance measures of 8,169,869 examinations in the National Breast Cancer Screening Program in Taiwan, 2004–2020
Source: BMC Med. 2023 Dec 15;21:497. doi: 10.1186/s12916-023-03217-7 (PMC10724902; doi:10.1186/s12916-023-03217-7)
Supplement: Supplementary file 2 — Additional file 2: Table S1. Clinical Demographics for Mammographic Screenings for Breast Cancer*. [file 12916_2023_3217_MOESM2_ESM.docx]

Additional file 2:

**Table S1. Clinical Demographics for Mammographic Screenings for Breast Cancer***

| **Characteristic** | | **2010-2020, Hospital** | | | |  | **2010-2020, Mobile** | | | |  |
| --- | --- | --- | --- | --- | --- | --- | --- | --- | --- | --- | --- |
|  |  | **Total** | **%** | **No. with SDCs** | **%** |  | **Total** | **%** | **No. with SDCs** | **%** | |
| **No. of women-visits** | | **3,762,883** |  | **18,372** |  |  | **3,681,576** |  | **12,002** |  | |
| **Age group, y** | |  |  |  |  |  |  |  |  |  | |
|  | 40-44 | 24,024 | **0.6** | 116 | **0.6** |  | 9289 | **0.3** | 22 | **0.2** | |
|  | 45-49 | 871,157 | **23.2** | 3897 | **21.2** |  | 704,087 | **19.1** | 2157 | **18.0** | |
|  | 50-54 | 890,687 | **23.7** | 4103 | **22.3** |  | 777,786 | **21.1** | 2399 | **20.0** | |
|  | 55-59 | 829,625 | **22.0** | 3949 | **21.5** |  | 835,665 | **22.7** | 2666 | **22.2** | |
|  | 60-64 | 676,952 | **18.0** | 3661 | **19.9** |  | 776,417 | **21.1** | 2731 | **22.8** | |
|  | 65-69 | 470,438 | **12.5** | 2646 | **14.4** |  | 578,332 | **15.7** | 2027 | **16.9** | |
|  |  |  |  |  |  |  |  |  |  |  | |
| **Screening service** | |  |  |  |  |  |  |  |  |  | |
|  | Prevalence screen | 1,513,688 | **40.2** | 9070 | **49.4** |  | 963,372 | **26.2** | 4114 | **34.3** | |
|  | Subsequent screen | 2,249,195 | **59.8** | 9302 | **50.6** |  | 2,718,204 | **73.8** | 7888 | **65.7** | |
|  |  |  |  |  |  |  |  |  |  |  | |
| **Menopausal status** | |  |  |  |  |  |  |  |  |  | |
|  | Premenopausal | 1,155,137 | **30.7** | 5808 | **31.6** |  | 1,008,949 | **27.4** | 3408 | **28.4** | |
|  | Postmenopausal | 2,607,746 | **69.3** | 12,564 | **68.4** |  | 2,672,627 | **72.6** | 8594 | **71.6** | |
|  |  |  |  |  |  |  |  |  |  |  | |
| **Breast density** | |  |  |  |  |  |  |  |  |  | |
|  | (A) Fatty breast | 145,887 | **3.9** | 442 | **2.4** |  | 164,872 | **4.5** | 307 | **2.6** | |
|  | (B) Scattered fibroglandular density | 812,750 | **21.6** | 3429 | **18.7** |  | 842,691 | **22.9** | 2590 | **21.6** | |
|  | (C) Heterogeneously dense | 2,230,498 | **59.3** | 12,065 | **65.7** |  | 1,938,954 | **52.7** | 7053 | **58.8** | |
|  | (D) Extremely dense | 573,635 | **15.2** | 2436 | **13.3** |  | 734,984 | **20.0** | 2050 | **17.1** | |
| **Family history of breast cancer** | | |  |  |  |  |  |  |  |  | |
|  | No | 3,469,104 | **92.2** | 16,305 | **88.7** |  | 3,469,309 | **94.2** | 10,994 | **91.6** | |
|  | Yes | 293,747 | **7.8** | 2067 | **11.3** |  | 212,229 | **5.8** | 1008 | **8.4** | |

*SDC denotes screen-detected cancer.
