# Supplementary material for: Performance measures of 8,169,869 examinations in the National Breast Cancer Screening Program in Taiwan, 2004–2020
Source: BMC Med. 2023 Dec 15;21:497. doi: 10.1186/s12916-023-03217-7 (PMC10724902; doi:10.1186/s12916-023-03217-7)

Additional file 3:

**Figure S1. Overall Survival Rates in Women with Breast Cancer Across the Two Time Periods by Age Group. (a) 40-49, (b) 50-54, (c) 55-59, (d) 60-64, (e) 65-69**

1. 40-49


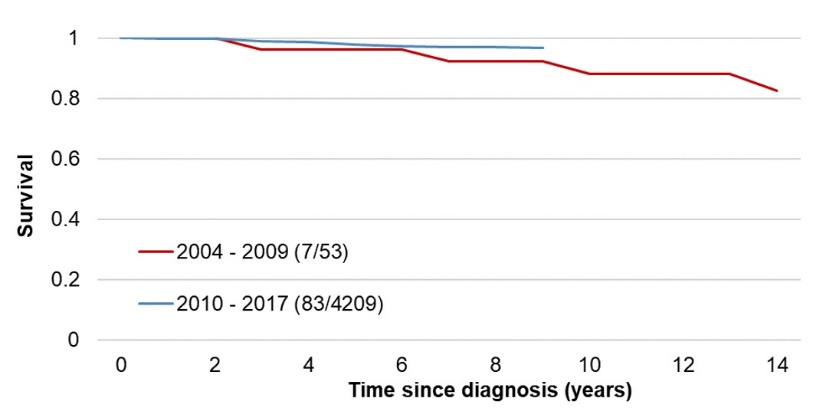


1. 50-54


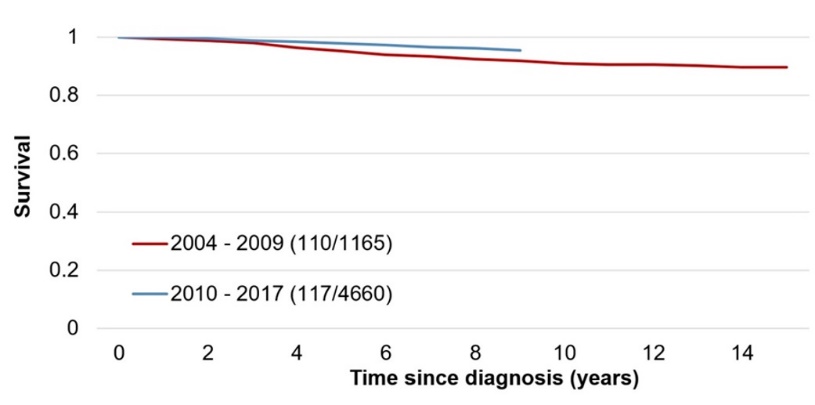


1. 55-59


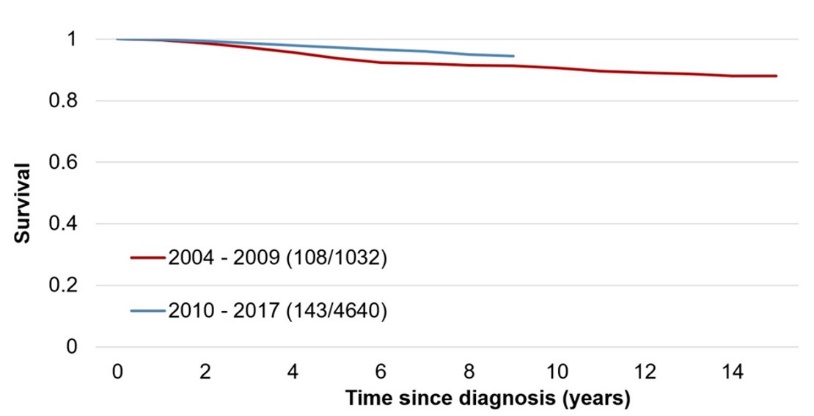


1. 60-64


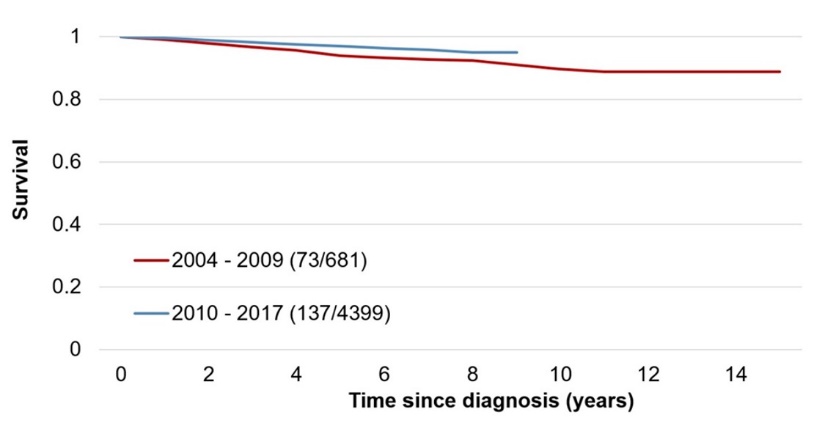


1. 65-69


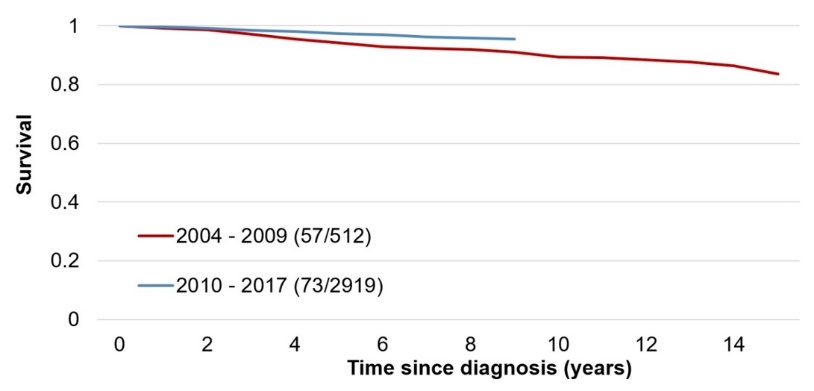

Supplement: Supplementary file 3 — Additional file 3: Figure S1. Overall Survival Rates in Women with Breast Cancer Across the Two Time Periods by Age Group. (a) 40-49, (b) 50-54, (c) 55-59, (d) 60-64, (e) 65-69. [file 12916_2023_3217_MOESM3_ESM.docx]
