# Supplementary material for: Performance measures of 8,169,869 examinations in the National Breast Cancer Screening Program in Taiwan, 2004–2020
Source: BMC Med. 2023 Dec 15;21:497. doi: 10.1186/s12916-023-03217-7 (PMC10724902; doi:10.1186/s12916-023-03217-7)
Supplement: Supplementary file 4 — Additional file 4: Table S2. Performance Measure for Mammographic Screenings Including Self-Reported Symptoms, 2010-2017. [file 12916_2023_3217_MOESM4_ESM.docx]

Additional file 4:

**Table S2. Performance Measure for Mammographic Screenings Including Self-Reported Symptoms,** **2010-2017***

|  | | **Hospital** | **Mobile** | **Subtotal** |
| --- | --- | --- | --- | --- |
| **Recall rate, % (95% CI)** | | **9.72**  **(9.69, 9.75)** | **7.58**  **(7.56, 7.61)** | **8.7**  **(8.68,8.72)** |
|  | No. of abnormal interpretations | 404,748 | 289,874 | 694,622 |
|  | Total no. of examinations | 4,162,802 | 3,822,990 | 7,985,792 |
|  |  |  |  |  |
| **CDR per 1000 examinations, No. (95% CI)** | | **7.42**  **(7.34, 7.51)** | **3.75**  **(3.68, 3.81)** | **5.66**  **(5.61,5.72)** |
|  | No. of cancers detected | 30,907 | 14,321 | 45,228 |
|  | Total no. of examinations | 4,162,803 | 3,822,990 | 7,985,793 |
|  |  |  |  |  |
| **PPV1, abnormal interpretations, % (95% CI)** | | **7.64**  **(7.55, 7.72)** | **4.94**  **(4.86, 5.02)** | **6.51**  **(6.45,6.57)** |
|  | No. of cancers detected | 30,907 | 14,321 | 45,228 |
|  | Initial BI-RADS category of 0,3,4, or 5 | 404,748 | 289,874 | 694,622 |
|  |  |  |  |  |
| **PPV2, biopsy recommended, % (95% CI)** | | **33.02**  **(32.72, 33.32)** | **30.86**  **(30.44, 31.28)** | **32.31**  **(32.06,32.55)** |
|  | No. of cancers detected | 30,907 | 14,321 | 45,228 |
|  | Final BI-RADS category of 4 or 5 | 93,592 | 46,407 | 139,999 |
|  |  |  |  |  |
| **PPV3, biopsy performed, % (95% CI)** | | **42.32**  **(41.96, 42.67)** | **39.43**  **(38.93, 39.93)** | **41.36**  **(41.07,41.65)** |
|  | No. of cancers detected | 30,907 | 14,321 | 45,228 |

*CI denotes confidence interval; BI-RADS denotes Breast Imaging Reporting & Data System; CDR denotes screen-detected cancer, 95% CI: 95% Confidence Interval.
