# Supplementary material for: Performance measures of 8,169,869 examinations in the National Breast Cancer Screening Program in Taiwan, 2004–2020
Source: BMC Med. 2023 Dec 15;21:497. doi: 10.1186/s12916-023-03217-7 (PMC10724902; doi:10.1186/s12916-023-03217-7)
Supplement: Supplementary file 5 — Additional file 5: Table S3. Characteristics of Breast Cancers Detected using Digital Mammographic Screening. [file 12916_2023_3217_MOESM5_ESM.docx]

Additional file 5:

**Table S3. Characteristics of Breast Cancers Detected using Digital Mammographic Screening ***

|  | | **2004-2009** | | **2010-2017*** | | | | | |
| --- | --- | --- | --- | --- | --- | --- | --- | --- | --- |
|  |  | **No.** | % | **Subtotal** | **%** | **Mobile** | **%** | **Hospital** | **%** |
| **No. of women with cancer** | | 3452 |  | 20,914 |  | 7611 |  | 13,303 |  |
|  | |  |  |  |  |  |  |  |  |
| **Tumor size, mm** | |  |  |  |  |  |  |  |  |
|  | DCIS | 630 | **21.01** | 2150 | **29.41** | 3981 | **31.28** | 6131 | **30.60** |
|  | 1-5 | 173 | **5.77** | 651 | **8.90** | 1171 | **9.20** | 1822 | **9.09** |
|  | 6-10 | 257 | **8.57** | 813 | **11.12** | 1423 | **11.18** | 2236 | **11.16** |
|  | 11-15 | 483 | **16.11** | 1276 | **17.45** | 2052 | **16.12** | 3328 | **16.61** |
|  | 16-20 | 417 | **13.90** | 887 | **12.13** | 1315 | **10.33** | 2202 | **10.99** |
|  | >20 | 1039 | **34.64** | 1534 | **20.98** | 2784 | **21.88** | 4318 | **21.55** |
|  |  |  |  |  |  |  |  |  |  |
| **Minimal cancer** | |  |  |  |  |  |  |  |  |
|  | Yes | 1850 | **56.08** | 13,515 | **67.26** | 4906 | **66.84** | 8609 | **67.51** |
|  | No | 1449 | **43.92** | 6578 | **32.74** | 2434 | **33.16** | 4144 | **32.49** |
|  |  |  |  |  |  |  |  |  |  |
| **Axillary lymph node status** | | |  |  |  |  |  |  |  |
|  | Negative | 1203 | **61.31** | 8049 | **72.60** | 3013 | **73.52** | 5036 | **72.06** |
|  | Positive | 759 | **38.69** | 3038 | **27.40** | 1085 | **26.48** | 1953 | **27.94** |
|  |  |  |  |  |  |  |  |  |  |
| **Cancer stage** | |  |  |  |  |  |  |  |  |
|  | 0 | 645 | **20.59** | 6349 | **32.25** | 2227 | **31.13** | 4122 | **32.89** |
|  | I | 1050 | **33.52** | 8024 | **40.76** | 3033 | **42.39** | 4991 | **39.83** |
|  | II | 995 | **31.77** | 3964 | **20.14** | 1467 | **20.50** | 2497 | **19.93** |
|  | III | 396 | **12.64** | 1195 | **6.07** | 385 | **5.38** | 810 | **6.46** |
|  | IV | 46 | **1.47** | 154 | **0.78** | 43 | **0.60** | 111 | **0.89** |
|  |  |  |  |  |  |  |  |  |  |

*DCIS denotes Ductal Carcinoma in situ.

*Most of the cancers were stage 0 or 1 (73.01%), minimal cancers, defined as DCIS or invasive cancers but smaller than 10 mm (67.26%), or node-negative (72.6%). Comparatively, in the mammograms collected from 2004 through 2009, 3452 indicated breast cancer, and of these, 630 (18.25%) were DCIS. Minimal cancers were found at a rate that was 1.2 times lower, but metastatic cancers were found at a rate that was higher by 1.47%.
