# Supplementary material for: Performance measures of 8,169,869 examinations in the National Breast Cancer Screening Program in Taiwan, 2004–2020
Source: BMC Med. 2023 Dec 15;21:497. doi: 10.1186/s12916-023-03217-7 (PMC10724902; doi:10.1186/s12916-023-03217-7)
Supplement: Supplementary file 6 — Additional file 6: Figure S2. The number of qualified radiologists and radiographers involved in the nationwide mammography screening program from 2004-2020. [file 12916_2023_3217_MOESM6_ESM.docx]

Additional file 6:

**Figure S2. The number of qualified radiologists and radiographers involved in the nationwide mammography screening program from 2004-2020**
