# Supplementary material for: Performance measures of 8,169,869 examinations in the National Breast Cancer Screening Program in Taiwan, 2004–2020
Source: BMC Med. 2023 Dec 15;21:497. doi: 10.1186/s12916-023-03217-7 (PMC10724902; doi:10.1186/s12916-023-03217-7)
Supplement: Supplementary file 7 — Additional file 7: Tables S4. Radiologists’ Performance Measures by Time Period. [file 12916_2023_3217_MOESM7_ESM.docx]

Additional file 7:

**Tables S4. Radiologists’ Performance Measures by Time Period**

|  | **2004-2009**  **Median (IQR)** | **2010-2020**  **Median (IQR)** |
| --- | --- | --- |
| **Recall rate, %** | 8.74  (5.76, 12.82) | 8.27  (6.42, 10.37) |
| **Cancer detection rate per 1000** | 4.33  (2.84, 5.85) | 4.02  (2.87, 5.25) |
| **PPV1, %** | 4.40  (2.74, 6.32) | 4.80  (3.69, 6.18) |
| **PPV2 (%)** | 33.33  (26.11, 42.99) | 27.56  (21.96, 33.33) |
| **PPV3 (%)** | 46.50  (39.79, 63.44) | 36.14  (29.38, 43,40) |
| **Sensitivity, %^†^** | 85.71  (80.00, 92.00) | 85.71  (79.41, 89.74) |
| **Specificity, %^†^** | 92.02  (87.83, 94.53) | 92.09  (89.91, 93.91) |
| **MR, %^†^** | 57.14  (47.11, 66.31) | 67.36  (61,11, 72.73)) |
| **TR, %^†^** | 52.38  (45.45, 63.16) | 73.64  (66.67, 80.73) |
| **NNR, %^†^** | 61.2  (53.94, 66.67) | 73.21  (66.67, 79.41) |
| **Mean invasive cancer size, mm (95% CI)^†^** | 20.44  (19.11, 22) | 18.57  (16.96, 19.98) |

*IQR denotes interquartile range; PPV denotes positive predictive value; MR denotes minimal cancer rate; TR denotes stage 0 or 1 cancer; NNR denotes negative node rate; and CI denotes confidence interval.

^†^Evaluated based on pre-2017 data.

^†^Based on the ACR BI-RADS 5th edition, our radiologists demonstrated good performance in sensitivity and specificity. Median sensitivity was 85.71% (IQR, 79.41%-89.74%) in both periods. Median specificity was 92.02% (IQR, 87.83%-94.53%) in the earlier period and 92.09% (IQR, 89.91%-93.91%) in the latter. Notably, the rate at which minimal cancer was found increased from 57.14% to 67.36% across the two periods.
