# Supplementary material for: Performance measures of 8,169,869 examinations in the National Breast Cancer Screening Program in Taiwan, 2004–2020
Source: BMC Med. 2023 Dec 15;21:497. doi: 10.1186/s12916-023-03217-7 (PMC10724902; doi:10.1186/s12916-023-03217-7)
Supplement: Supplementary file 8 — Additional file 8: Table S5. Radiologists’ Performance Measures by Screening Year. [file 12916_2023_3217_MOESM8_ESM.docx]

Additional file 8:

**Table S5. Radiologists’ Performance Measures by Screening Year**

|  | **2004** | **2005** | **2006** | **2007** | **2008** | **2009** |
| --- | --- | --- | --- | --- | --- | --- |
| **Recall rate, % (IQR)** | 1.19  (1.19, 1.19) | 6.50  (1.19, 15.09) | 7.45  (5.15, 12.00) | 7.78  (4.70, 11.17) | 7.42  (4.5, 11.23) | 8.21  (5.46, 12.19) |
| **Cancer detection rate per 1000, No. (IQR)** | 0  (0, 0) | 3.66  (1.76, 5.30) | 3.88  (2.28, 4.90) | 3.88  (1.96, 6.39) | 3.76  (2.35, 5.57) | 4.61  (3.35, 6.56) |
| **PPV1, % (IQR)** | 2.47  (2.00, 2.94) | 2.79  (0.87, 3.39) | 2.79  (1.19, 5.60) | 3.9  (2.84, 5.43) | 3.97  (2.48, 5.50) | 4.38  (2.99, 5.79) |
| **PPV2, % (IQR)** |  | 24.32  (18.92, 33.90) | 38.18  (21.28, 39.47) | 27.79  (22.58, 31.75) | 28.57  (21.88, 41.38) | 29.22  (21.74, 34.88) |
| **PPV3, % (IQR)** |  | 18.42  (18.42, 18.42) | 42  (42, 42) | 37.86  (35.71, 40.00) | 42.86  (36.37, 58.39) | 42.68  (34.99, 45.04) |
| **Sensitivity, % (IQR)^†^** |  | 100  (100, 100) | 95.45  (68.75, 100.00) | 83.33  (80.00, 89.47) | 88.89  (85.71, 96.30) | 82.84  (73.91, 92.59) |
| **Specificity, % (IQR)^†^** | 98.8  (98.8, 98.8) | 93.74  (85.44, 99.08) | 92.92  (88.36, 95.23) | 92.61  (89.10, 95.39) | 92.92  (88.76, 95.84) | 92.21  (88.53, 94.99) |
| **MR, % (IQR)^†^** |  | 56.08  (53.33, 58.82) | 47.62  (47.62, 47.62) | 67.54  (66.67, 68.42) | 56.52  (50.00, 66.67) | 50.12  (42.86, 61.11) |
| **TR, % (IQR)^†^** |  | 50.00  (33.33, 66.67) | 47.37  (47.37, 47.37) | 53.51  (33.33, 73.68) | 57.27  (54.55, 62) | 60.00  (52.94, 65.00) |
| **NNR, % (IQR)^†^** |  |  |  |  | 27.78  (27.78, 27.78) | 71.74  (60.87, 82.61) |
| **Mean invasive cancer size, mm (IQR)^†^** |  |  | 19.81  (19.81, 19.81) |  | 19.61  (18.94, 21.12) | 19.47  (17.44, 19.84) |

|  | **2010** | **2011** | **2012** | **2013** | **2014** | **2015** |
| --- | --- | --- | --- | --- | --- | --- |
| **Recall rate, % (IQR)** | 7.86  (5.55, 11.8) | 8.76  (6.77, 11.34) | 8.32  (6.25, 11.15) | 7.78  (6.33, 9.60) | 7.61  (6.04, 9.40) | 7.76  (6.00, 9.33) |
| **Cancer detection rate per 1000, No. (IQR)** | 3.70  (2.49, 5.24) | 3.84  (2.61, 5.06) | 3.91  (2.80, 4.95) | 3.98  (2.84, 5.03) | 3.94  (2.52, 5.32) | 3.81  (2.58, 5.58) |
| **PPV1, % (IQR)** | 3.88  (2.68, 5.14) | 3.67  (2.49, 5.51) | 4.24  (3.06, 5.37) | 4.65  (3.24, 5.95) | 4.78  (3.18, 5.86) | 4.93  (3.58, 6.72) |
| **PPV2, % (IQR)** | 22.65  (17.49, 27.86) | 22.47  (17.41, 30.04) | 23.33  (18.18, 29.03) | 22.22  (17.72, 31.34) | 26.21  (18.75, 34.62) | 26.23  (17.13, 32.65) |
| **PPV3, % (IQR)** | 31.25  (25.00, 37.84) | 33.33  (28.13, 44) | 32.50  (26.10, 39.15) | 30.01  (24.69, 40.74) | 33.33  (26.45, 41.71) | 34.62  (25.35, 42.55) |
| **Sensitivity, % (IQR)^†^** | 83.33  (75.86, 90.57) | 87.50  (83.33, 91.30) | 86.36  (78.95, 92.00) | 86.93  (81.25, 90.63) | 86.60  (81.25, 93.33) | 84.85  (80, 88.89) |
| **Specificity, % (IQR)^†^** | 92.58  (88.48, 94.80) | 91.50  (89.03, 93.56) | 92.05  (89.33, 94.06) | 92.60  (90.60, 93.94) | 92.76  (91.05, 94.46) | 92.71  (91.11, 94.33) |
| **MR, % (IQR)^†^** | 66.67  (63.64, 72.41) | 68.59  (60, 74.07) | 62.07  (55.56, 75.76) | 68.75  (61.90, 80.00) | 70.82  (65.94, 76.47) | 70.59  (61.90, 78.49) |
| **TR, % (IQR)^†^** | 73.33  (66.67, 80) | 74.17  (68.03, 82.55) | 76.33  (68.14, 81.85) | 76.19  (64.71, 88.00) | 73.33  (63.16, 80.77) | 75.00  (66.67, 82.35) |
| **NNR, % (IQR)^†^** | 70.83  (66.67, 73.33) | 63.77  (54.17, 72.28) | 71.05  (66.67, 72.22) | 68.75  (63.16, 72.22) | 70.59  (66.67, 78.57) | 76.47  (75.00, 78.95) |
| **Mean invasive cancer size, mm (IQR)^†^** | 18.33  (18.06, 19.26) | 18.57  (17.50, 19.43) | 17.70  (17.03, 19.42) | 18.37  (17.43, 19.57) | 18.20  (17.67, 20.56) | 18.30  (16.70, 19.21) |

|  | **2016** | **2017** | **2018** | **2019** | **2020** |
| --- | --- | --- | --- | --- | --- |
| **Recall rate, % (IQR)** | 7.49  (6.08, 9.70) | 7.64  (6.14, 9.49) | 7.56  (6.01, 9.34) | 7.61  (6.08, 9.03) | 7.76  (6.29, 9.14) |
| **Cancer detection rate per 1000, No. (IQR)** | 4.02  (2.93, 5.69) | 4.50  (3.27, 5.95) | 4.41  (3.09, 6.07) | 3.7  (2.65, 4.99) | 3.73  (2.7, 5.36) |
| **PPV1, % (IQR)** | 5.27  (3.75, 6.90) | 5.56  (3.90, 7.14) | 5.71  (3.88, 7.76) | 4.72  (3.70, 6.18) | 4.80  (3.43, 6.26) |
| **PPV2, % (IQR)** | 27.96  (20.00, 35.82) | 28.57  (21.21, 36.36) | 29.27  (21.05, 38.24) | 24.17  (18.55, 30.55) | 25.19  (19.44, 32.43) |
| **PPV3, % (IQR)** | 35.29  (25.00, 44.19) | 36.36  (28.13, 44.44) | 36.00  (27.03, 41.94) | 29.41  (23.73, 35.48) | 30.00  (25.00, 38.10) |
| **Sensitivity, % (IQR)^†^** | 83.49  (77.78, 90.91) | 86.52  (80.00, 93.33) |  |  |  |
| **Specificity, % (IQR)^†^** | 92.94  (90.83, 94.32) | 92.79  (91.00, 94.40) |  |  |  |
| **MR, % (IQR)*** | 66.26  (58.44, 68.99) | 66.67  (58.82, 75.00) |  |  |  |
| **TR, % (IQR)^†^** | 75.6  (68.18, 83.33) | 81.25  (72.96, 85.70) |  |  |  |
| **NNR, % (IQR)^†^** | 80  (72.22, 81.25) | 77.73  (65.31, 88.92) |  |  |  |
| **Mean invasive cancer size, mm (IQR)^†^** | 17.94  (16.69, 19.27) | 17.29  (15.44, 18.91) |  |  |  |

*IQR denotes interquartile range; PPV denotes positive predictive value; MR denotes denotes minimal cancer rate; TR denotes stage 0 or 1 cancer; NNR denotes negative node rate; and CI denotes confidence interval.

^†^ Evaluated based on pre-2017 data.
