# Supplementary material for: Performance measures of 8,169,869 examinations in the National Breast Cancer Screening Program in Taiwan, 2004–2020
Source: BMC Med. 2023 Dec 15;21:497. doi: 10.1186/s12916-023-03217-7 (PMC10724902; doi:10.1186/s12916-023-03217-7)
Supplement: Supplementary file 9 — Additional file 9: Table S6. Performance Measures of Digital Mammographic Screenings for Breast Cancer in 2010-2020 by age group. [file 12916_2023_3217_MOESM9_ESM.docx]

Additional file 9:

**Table S6. Performance Measures of Digital Mammographic Screenings for Breast Cancer in 2010-2020 by age group*.**

| **Measure** | **2010-2020** | | |
| --- | --- | --- | --- |
|  | **<50** | $\boldsymbol{\geq}$**50** | **Subtotal** |
| **Recall rate, %** | **9.86**  **(9.81, 9.9)** | **7.68**  **(7.66, 7.7)** | **8.15**  **(8.13, 8.17)** |
| Total no. of examinations | 158,566 | 448,222 | 606,788 |
| No. of abnormal interpretation | 1,608,553 | 5,835,888 | 7,444,441 |
|  |  |  |  |
| **CDR per 1000 examinations, No.** | **3.97**  **(3.88, 4.07)** | **4.25**  **(4.19, 4.3)** | **4.19**  **(4.14, 4.23)** |
| No. detecting cancer | 6,390 | 24,775 | 31,165 |
| Total no. of examinations | 1,608,553 | 5,835,888 | 7,444,441 |
|  |  |  |  |
| **PPV1, abnormal interpretations, %** | **4.03**  **(3.93, 4.13)** | **5.53**  **(5.46, 5.59)** | **5.14**  **(5.08, 5.19)** |
| No. of mammograms detecting cancer | 6,390 | 24,775 | 31,165 |
| Initial BI-RADS category of 0, 3, 4, or 5 | 158,566 | 448,222 | 606,788 |
|  |  |  |  |
| **PPV2, biopsy recommended, %** | **20.81**  **(20.36, 21.26)** | **30.58**  **(30.27, 30.9)** | **27.9**  **(27.63, 28.16)** |
| No. of mammograms detecting cancer | 6,390 | 24,775 | 31,165 |
| Final BI-RADS category of 4 or 5 | 30,707 | 81,010 | 111,717 |
|  |  |  |  |
| **PPV3, biopsy performed, %** | **26.6**  **(26.04, 27.15)** | **40.5**  **(40.11, 40.89)** | **36.58**  **(36.26, 36.9)** |
| No. of mammograms detecting cancer | 6,390 | 24,775 | 31,165 |
| Final BI-RADS category of 4 or 5 with biopsy | 24,026 | 61,172 | 85,198 |

* Numbers in parentheses are 95% CIs; CDR, cancer detection rate; and PPV, positive predictive value
