# Supplementary material for: Performance measures of 8,169,869 examinations in the National Breast Cancer Screening Program in Taiwan, 2004–2020
Source: BMC Med. 2023 Dec 15;21:497. doi: 10.1186/s12916-023-03217-7 (PMC10724902; doi:10.1186/s12916-023-03217-7)
Supplement: Supplementary file 10 — Additional file 10: Figure S3. Distribution of screening population followed by age in 2 periods. [file 12916_2023_3217_MOESM10_ESM.docx]

Additional file 10:

**Figure S3. Distribution of screening population followed by age in 2 periods**

Distribution of screening population followed by age in the period 2004-2009

Distribution of screening population followed by age in the period 20010-2020
